# The Management and Outcomes of Septic Shock Among Surgical Patients at the Jimma University Medical Center, Jimma, Ethiopia: A Prospective Study

**DOI:** 10.7759/cureus.67723

**Published:** 2024-08-25

**Authors:** Langa James Oriho, Wongel Tena Shale, Samuel Tesfaye Woldemariam

**Affiliations:** 1 Department of Surgery, College of Public Health and Medical Sciences, Jimma University, Jimma, ETH; 2 Department of surgery, College of Public Health and Medical Sciences, Jimma University, Jimma, ETH

**Keywords:** septic shock in ethiopia, septic shock management, critical care, sepsis, septic shock

## Abstract

Background and objective

Sepsis and septic shock are major healthcare issues in surgical patients admitted to the surgery ward or ICU, affecting millions of people worldwide annually, with a mortality rate between one in three and one in six of those affected. The incidence and mortality of sepsis vary greatly by region, with the highest prevalence in Sub-Saharan Africa, Oceania, South Asia, East Asia, and Southeast Asia. Of all sepsis cases in 2017, 33.1 million people suffered from ill health due to underlying infectious diseases, and 15.8 million suffered from underlying injuries or non-communicable diseases.

Methods

This prospective observational study was conducted at the Jimma University Medical Centre (JUMC) in Jimma town in southwest Ethiopia, from April 2023 to October 2023. All surgical patients aged ≥15 years who presented with or developed septic shock at the Jimma University Medical Centre were included.

Results

The study involved a total of 61 patients. The median age of the patients was 45 years [interquartile range (IQR): 40-60 years], and 77% (n=47) of the patients were male. The most frequent source of infection in this study was community-acquired infection (83.3%, n=49). The most common focus of sepsis was the intra-abdominal infection of the digestive system (82%, n=50). Lactate level testing and blood cultures before administering antibiotics were not done for all septic shock patients. Source control surgery was performed in 52.5% (n=32) of patients after developing septic shock, and 84.4% (n=27) of surgeries were performed within 24 hours. The 30-day mortality rate was 80.3%, with an ICU mortality rate of 78.94%. The median length of stay in the ICU was three days (IQR: 1-5 days), and the median length of hospital stay was six days (IQR: 2-15 days).

Conclusions

The mortality rate in our cohort was higher compared to that in studies from high-income and low-income countries. There was poor adherence and compliance with the Surviving Sepsis Campaign (SSC) (the one-hour bundle) guidelines. The length of stay in hospitals and ICUs was lower compared to studies from high-income countries due to the high early mortality rates.

## Introduction

Septic shock is a serious healthcare issue that affects millions each year, with up to one in six people dying from it [1]. Early identification of septic shock is possible in a subset of sepsis in which underlying circulatory and cellular metabolism abnormalities are profound enough to substantially increase mortality. Executing proper management in the first few hours of its development can lead to better results [1]. Sepsis is defined as a “life-threatening condition caused by a dysregulated host response to infection, resulting in organ dysfunction” [2]. Septic shock is defined as “circulatory, cellular, and metabolic abnormalities in septic patients, presenting as fluid-refractory hypotension requiring vasopressor therapy with associated tissue hypoperfusion (lactate >2 mmol/L)”. The term severe sepsis is no longer used [2,3].

In 2017, 48.9 million cases of sepsis [95% uncertainty interval (UI): 38.9-62.9] were reported worldwide, and 11 million deaths (10.1-12.0 million), of which 19% were sepsis-related and accounted for 7% (18.2-21.4 million) of global death toll [4]. Although sepsis can affect anyone in any geographical region, there are large regional variations in its incidence and mortality, with the highest incidence reported in low- and middle-income countries (LMICs). Additionally, managing sepsis is expensive, with the average hospital-wide cost estimated at over $32,000 per patient. However, these estimates are based almost entirely on data from high-income countries (HIC) [4]. The incidence and mortality of sepsis vary greatly by region, with the highest prevalence in Sub-Saharan Africa, Oceania, South Asia, East Asia, and Southeast Asia.

Of all sepsis cases reported in 2017, 33.1 million people suffered from ill health due to underlying infectious diseases, and 15.8 million occurred in people suffering from underlying injuries or non-communicable diseases [4]. A study from Addis Ababa, Ethiopia aimed to assess the prevalence and outcome of sepsis and septic shock in ICUs. The prevalence of sepsis and septic shock was found to be 26.5 per 100 ICU admissions. Based on the Third International Consensus Definitions for Sepsis and Septic Shock (SEPSIS-3), 15.1% of patients had sepsis, and 8.9% of patients had septic shock during ICU admission [5]. Accurate tracking of sepsis incidence and outcome in LMICs is limited due to changing definitions, lack of diagnostic codes and health records, and understaffing. Improving sepsis treatment in LMIC requires conducting prospective outcomes studies to establish appropriate definitions, scoring systems, and treatment guidelines [6].

Regarding diagnosis, various screening tools are available to identify sepsis it early on. They involve either manual methods or the electronic health record. There is variation in the diagnostic accuracy of these tools; while most have not been proven to be very accurate, a few have been linked to improvements in care [1,7-8]. Several tools are used for screening of sepsis/septic shock, such as systemic inflammatory response syndrome (SIRS) criteria; vital signs; signs of infection; quick Sequential Organ Failure Score (qSOFA) or Sequential Organ Failure Assessment (SOFA) criteria; National Early Warning Score (NEWS); or Modified Early Warning Score (MEWS) [1,9-10].

Evaluation of the patient in septic shock begins with assessing the adequacy of their airway and ventilation. Severely obtunded patients and those whose work of breathing is excessive require intubation and ventilation to prevent respiratory collapse. Early vasopressors are a major management component, as delays are associated with increased mortality, and norepinephrine has a "fluid-like" effect via recruiting unstressed volume. Early goal-directed therapy is essential. The Surviving Sepsis Campaign (SSC) has updated treatment guidelines with a most recent goal for care within the early hours [3]. This study aimed to assess the management and outcomes of septic shock among surgical patients at the Jimma University Medical Centre (JUMC) from April 2023 to October 2023, to identify the focus of infection among surgical patients with septic shock, to assess adherence to surviving sepsis guidelines in managing surgical patients with septic shock, and to determine the outcome (30-day mortality and postoperative complications) of patients with septic shock.

## Materials and methods

Study design

This prospective observational study was conducted at JUMC from April 2023 to October 2023. We prospectively recruited surgical patients aged ≥15 years who presented with or developed septic shock according to the SEPSIS-3 criteria at JUMC. The inclusion criteria were as follows: surgical patients aged ≥15 years who presented with septic shock according to the Sepsis-3 definition and criteria, those surgical patients who developed septic shock as a complication throughout their stay at the hospital, and patients from other departments in the hospital who presented or/developed septic shock due to surgical condition and surgery consulted department. Pediatric patients aged <15 years presenting with septic shock, patients who developed septic shock due to obstetric or gynecological conditions, and surgical patients developing septic shock after being discharged from the hospital were excluded. All participating patients were followed up until 30 days of stay. Patients discharged before 30 days were monitored for the outcome at 30 days using their follow-up records and phone calls.

Sample size calculation

A single population proportion sample size determination formula was employed. The sample size (n) was determined using the following statistical formula for a single proportion: n=Z^2^*P* (1-P)/d^2^, where n=sample size, d=margin of error between the sample and the population 5%, Z=Z score for 95% confidence interval (CI) (1.96), and p=8.9% referred prevalence of the study done in Addis Ababa, Ethiopia [5]. The calculated sample size was n=125; unfortunately, only 61 patients in the study met the inclusion criteria throughout the study period. 

Operational definitions

Sepsis

Sepsis was defined as a life-threatening organ dysfunction caused by a dysregulated host response to infection. Organ dysfunction was determined as an acute change in total SOFA score of 2 points consequent to the infection. The baseline SOFA score was assumed to be 0 in patients not known to have preexisting organ dysfunction. 

Septic Shock

This was defined as a clinical construct of sepsis with persisting hypotension requiring vasopressors to maintain a mean arterial pressure (MAP) of 65 mmHg and having a serum lactate level >2 mmol/L (18 mg/dL) despite adequate volume resuscitation.

Community-Onset Sepsis

This condition was defined as the onset of sepsis within two days of hospital admission, where the date of admission was counted as hospital day one.

Hospital-Onset Sepsis

This was defined as the onset of sepsis on hospital day three or later, counting the admission date as hospital day one.

Postoperative Complications/Outcomes

According to the Clavien-Dindo classification, this is a deviation from the normal postoperative course, meaning that the severity ranges from non-life-threatening complications with no lasting disability to fatal outcomes.

Study variables

Dependent variables included outcomes (postoperative complications, 30-day mortality) and hospital/ICU length of stay. Independent variables were sociodemographic factors (age, sex, marital status, residence, and educational status), and comorbidities. The biomedical factors (anemia), cause of septic shock, source of infection, comorbidities, time of presentation, arrival, and time of intervention, complications, sepsis screening tool [Modified Sequential Organ Failure Assessment (mSOFA) score], and management steps of septic shock (ventilator support, fluid resuscitation, vasopressor, antibiotics administration, culture/sensitivity, surgical intervention, blood products transfusion, glycemic control, stress ulcer/deep vein thrombosis prophylaxis, counseling) were also documented.

Data collection

A semi-structured checklist comprising the variables measured was designed, developed, and utilized based on previous literature with necessary modifications and incorporations as required. Meanwhile, research assistants were trained by the principal investigator to collect the data. The principal investigator also controlled the accuracy, completeness, and consistency of the data collected during the observational prospective study.

Data processing and analysis

The data from the checklist was entered into EpiData Manager (v4.6.0.6) software and exported and analyzed using SPSS Statistics Version 27 (IBM Corp., Armonk, NY). Descriptive statistics such as percentages, median, and interquartile range (IQR) were calculated to summarize the data. The results were also presented in the form of figures and tables. 

Ethical approval

This study was approved by the Institutional Review Board of Jimma University Medical Centre (approval no: JUIH/IRB/538/23). Patients' consents were taken and honored, and confidentiality of medical records including patient name and demographic data was maintained. This research was observational, and hence normal patient management was done according to existing hospital management guidelines, and no tests or procedures were performed as part of the research.

## Results

**Figure 1:**
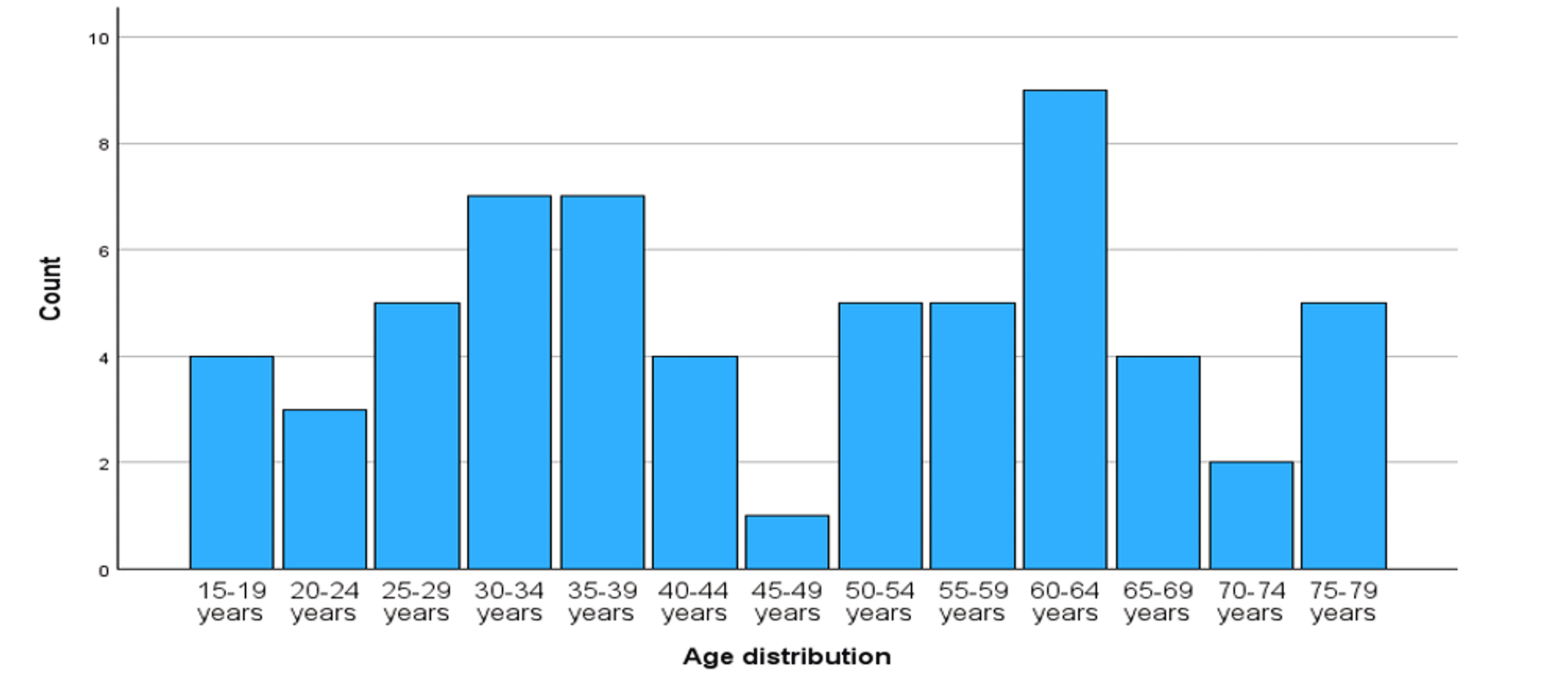
Age distribution among surgical patients with septic shock

**Figure 2:**
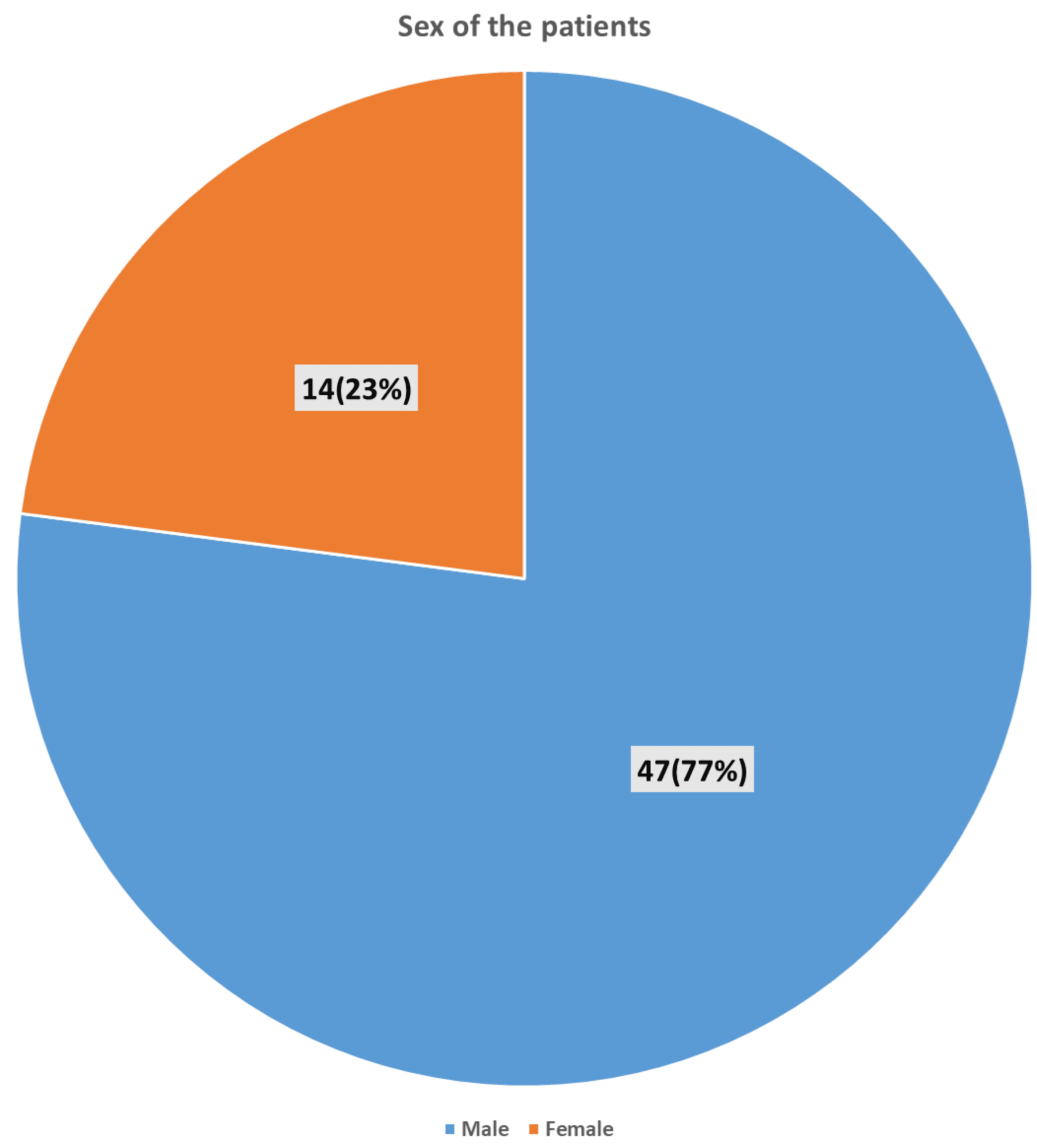
Gender distribution among surgical patients with septic shock

We included 61 patients in the study. The median age of patients was 45 years (IQR: 40-60 years), as seen in Figure 1, and 77% (n=47) of the patients were male (Figure 2). Regarding education level, 42% of patients could at least read and write, and 57.4% were illiterate. The majority, 68.9% of septic shock patients, reported being professionally active and 31.1% were unemployed.

The most frequent source of infection in this study was community-onset sepsis (83.3%, n=49). The most common focus of infection in septic shock in surgical patients was the intra-abdominal infection of the digestive system (82%, n=50), followed by the respiratory system (11.5%, n=7), genitourinary system (3.3%, n=2), and musculoskeletal system (3.3%, n=2) (Table 1). Of the 61 patients in this study, 29.5% (n=18) had comorbidities, and 9.8% (n=6) had two or more comorbidities. The most common comorbid condition was malignancy (11.47%, n=7) (Table 2). Only 13.1% (n=8) of patients in the study were tested for HIV. One patient was positive, and seven were negative for HIV. The rate of ICU admission was 62.3% (n=38), and the median Modified Sequential Organ Failure Assessment (mSOFA) score at admission was 10.

**Table 1:** General characteristics of surgical patients with septic shock ICU: intensive care unit; IQR: interquartile range

Variable		Value
Gender, n (%)	Male	47 (77%)
	Female	15 (23%)
Marital status, n (%)	Married	48 (78.7%)
	Single	12 (19.7%)
	Divorced	1 (1.6%)
	Widowed	0
	Other	0
Educational status, n (%)	Able to read and write	26 (42.6%)
	Unable to read and write	35 (57.4%)
Employment status, n (%)	Employed	42 (68.9%)
	Unemployed	29 (31.1%)
Residential area, n (%)	Urban	8 (13.1%)
	Rural	53 (86.9%)
Source of infection, n (%)	Community-onset sepsis	49 (80.3%)
	Hospital-onset sepsis	10 (16.4%)
	ICU-onset sepsis	2 (3.3%)
Focus of sepsis, n (%)	Intra-abdominal	50 (82%)
	Genitourinary system	2 (3.3%)
	Musculoskeletal	2 (3.3%)
	Respiratory	7 (11.5%)
ICU length of stay, days, median (IQR)		3 (1–5)
Hospital length of stay, days, median (IQR)		6 (2–15)
30-day outcome, n (%)	Survival	12 (19.7%)
	Death	49 (80.3%)

**Table 2:** Comorbidities in surgical patients with septic shock CKD: chronic kidney disease; IHD: ischemic heart disease; PAD: peripheral arterial disease; TB: tuberculosis

Comorbidity	Frequency
Malignancy	7
Hypertension	6
Community-acquired pneumonia	2
PAD	2
CKD	2
IHD	1
Asthma	1
TB	2
Mania	1
Adult-onset malnutrition	1
≥2 comorbidities	6

Management of septic shock

Of 61 surgical patients with septic shock, 68% (n=42) required mechanical ventilator support, 38 patients received mechanical ventilator support on admission to the ICU, and only two patients received it in the operating room (due to the limited number of mechanical ventilators in the hospital ICU, the patients had ventilatory support from the anesthesia machine while awaiting the mechanical ventilator to be available).

Regarding fluid management, 55.7% (n=34) of patients received 30 ml/kg of crystalloid within three hours, 42.6% (n=26) received more than 30 ml/kg of crystalloid, and only one patient (1.6%) received crystalloid infusion less than 30 ml/kg in three hours (Table 3). Serum lactate level measurement was not done for all the patients.

**Table 3:** Management steps for surgical patients with septic shock DVT: deep vein thrombosis

Management steps		Outcome (30 days)		Total, n (%)
		Survival, n	Death, n	
Respiratory management	Mechanical ventilator	6	36	42 (68.9%)
	Face-mask oxygen	1	9	10 (16.4%)
	Intranasal oxygen	5	3	8 (13.1%)
	No respiratory support	0	1	1 (1.6%)
Fluid administration	30 ml/kg within 3 hours	10	24	34 (55.7%)
	>30 ml/kg within 3 hours	2	24	26 (42.6)
	<30 ml/kg within 3 hours	0	1	1 (1.6%)
Vasopressor type	Adrenaline	12	46	58 (95.1%)
	Not administered	0	3	3 (4.9%)
Other vasopressor added	Yes (dopamine was added in 3 patients and noradrenaline replaced adrenaline in 1 patient)	1	4	5 (8.2%)
	No	11	41	52 (85.2%)
Microbial culture samples collected	Yes	2	9	11 (18%)
	No	10	40	50 (82%)
Blood transfusion	Number of patients transfused according to the transfusion trigger of 70 g/L	1	4	5 (8.2%)
	The number of patients transfused was not per the transfusion trigger of 70 g/L due to the presence of comorbidities	10	34	44 (72.1%)
	Number of patients who did not receive blood transfusion	1	11	12 (19.7%)
Stress ulcer prophylaxis	Administered	8	30	38 (63%)
	Not administered due to the unavailability of medication	4	19	23 (37.7%)
DVT prophylaxis	Administered	5	19	24 (39.3%)
	Not administered because of unavailability of medication	7	30	37 (60.7%)
Steroids	Administered	4	23	27 (44.3%)
	Not administered due to the unavailability of medication	8	26	34 (55.7%)
Glycemic control (insulin administered)	Performed	2	3	5 (8.2%)
	Not performed	10	46	56 (91.8)

The initial vasopressor was administered in 95% (n=58, adrenaline) of patients with septic shock, whereas it was not administered in 4.9% (n=3) due to unavailability. In 82% (n=50) of patients, vasopressors were initiated within one to six hours after the diagnosis of septic shock. In four patients, vasopressor (adrenaline) was combined with dopamine to maintain MAP; in one patient, adrenaline was replaced with noradrenaline to maintain MAP. Microbial culture samples were collected in 18% (n=11) of patients, whereas cultures were not performed in the remaining 82% (n=50) of patients for various reasons. Five patients had no microbial growth, and six had microbial growth: three Escherichia coli and one Acinetobacter species., one Pseudomonas species., and one Staphylococcus aureus. All samples for cultures and sensitivity in this study were collected after empirical antibiotics were given.

All 61 patients in the study received empiric antibiotics, and 95% (n=58) of patients received antibiotics within one hour. The most frequent antibiotic combination was ceftriaxone/metronidazole (55.7%, n=34), followed by ceftazidime/vancomycin/metronidazole (18%, n=11) and ceftriaxone/vancomycin/metronidazole 11.5% (n=7). Only one patient's antibiotics were adapted after culture and sensitivity results.

Source control surgery was performed in 52.5% (n=32) of patients after developing septic shock, and 84.4% (n=27) of surgeries were performed within 24 hours of presentation. Of note, 47.5% (n=29) of patients did not undergo surgery after septic shock due to the following reasons: 11 patients had septic shock immediately after surgery, seven patients were not optimized for surgery, five were moribund patients not fit for surgery, and five patients were scheduled for a second-look surgery and died before surgery, one patient did not receive surgery for an unknown reason. Reoperation or second-look surgery was performed in 23% (n=14) of the patients. As for postoperative complications, 45.9% (n=28) of patients developed postoperative complications in the ward due to the intervention or previous interventions. The most common postoperative complication was surgical site infection at 16.39% and hospital-acquired pneumonia at 16.39% (Table 4).

**Table 4:** Postoperative complications in surgical patients with septic shock

Postoperative complication	Frequency
Hospital-acquired pneumonia	10
Surgical site infection	10
Hypernatremia	4
Abdominal wound dehiscence	3
Acute kidney injury	3
Anemia	3
Hypoalbuminemia	3
Hypokalemia	2
Hypochloremia	2
Aspiration pneumonia	2
Bed sores	2
Postoperative intussusception	1
High output fistula	1
Intra-abdominal collection	1
Heart failure	1
Tracheoesophageal fistula	1
≥2 postoperative complications	16

Corticosteroids were administered to 44.3% (n=27) of patients, while deep vein thrombosis (DVT) prophylaxis was given to 39.3% (n=24) of patients, and stress ulcer prophylaxis to 62.3% (n=38). All patients or their relatives received some advice about the prognosis of septic shock and the limits of support.

Outcomes of septic shock

**Figure 3:**
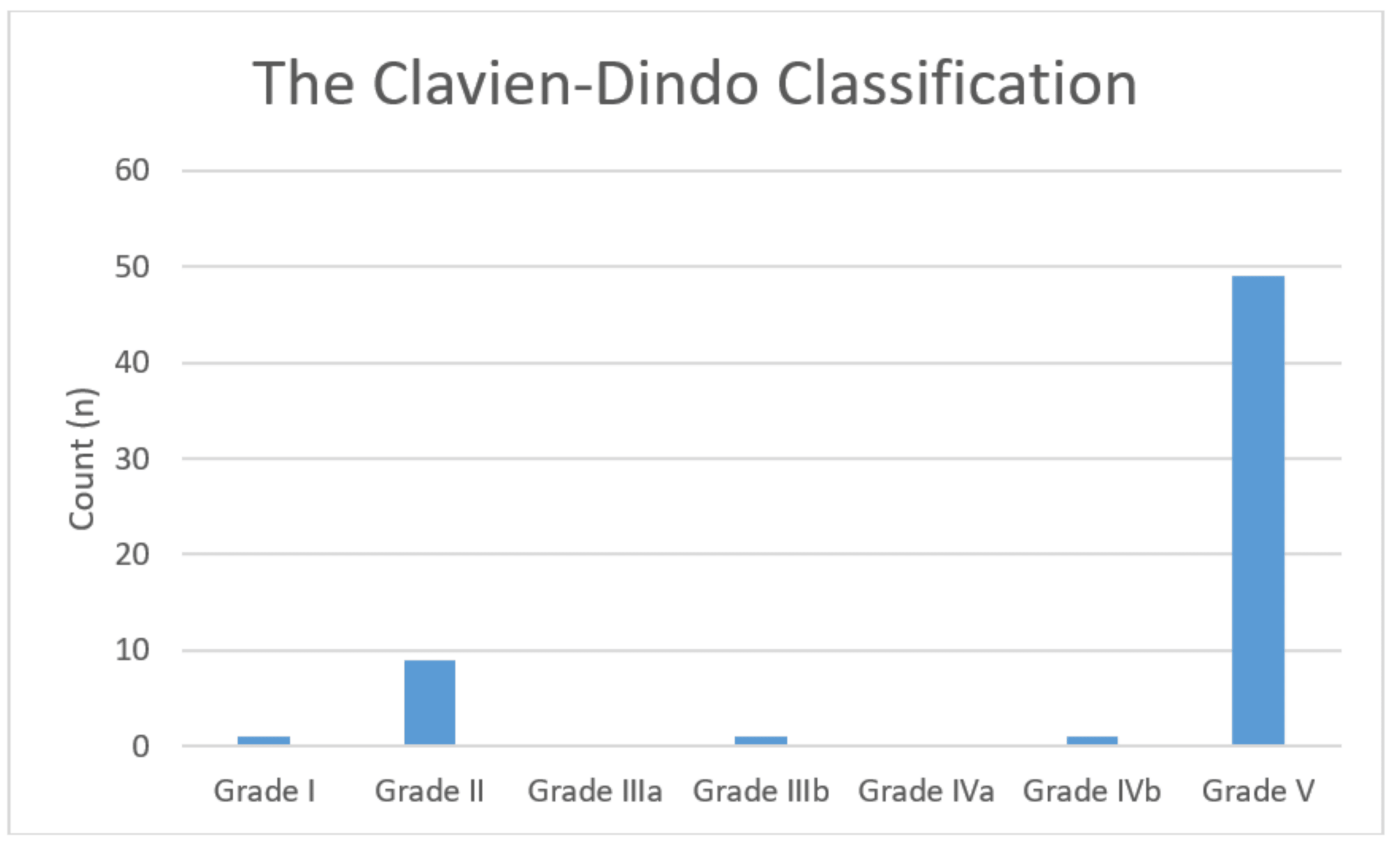
The Clavien-Dindo classification of septic shock in surgical patients Grade I: any deviation from the normal postoperative course without the need for pharmacological treatment or surgical, endoscopic, and radiological interventions. The allowed therapeutic regimens are drugs such as antiemetics, antipyretics, analgesics, diuretics, electrolytes, and physiotherapy. This grade also includes wound infections opened at the bedside. Grade II: requiring pharmacological treatment with drugs other than those allowed for grade I complications. Blood transfusions and total parenteral nutrition are also included. Grade III: requiring surgical, endoscopic, or radiological intervention; IIIa: intervention not under general anesthesia; and IIIb: intervention under general anesthesia. Grade IV: life-threatening complication (including CNS complications) requiring IC/ICU management; IVa: single organ dysfunction (including dialysis); and IVb: multi-organ dysfunction. Grade V: death of the patient CNS: central nervous system; ICU: intensive care unit

Septic shock was diagnosed in 61 patients, of which 49 (80.3%) died within 30 days, with an ICU mortality rate of 78.94% (Table 1). Most of the deaths were due to community-onset sepsis (Table 5). The Clavien-Dindo classification of the cohort was as follows: Grade V: 80.3%, n=49; Grade II: 14%, n=9; Grade I: 1.6%, n=1; Grade IIIa: 0%, n=0); Grade IIIb: 1.6%, n=1; Grade IVa: 0%, n=0; and Grade IVb: 1.6%, n=1; (Figure 3). The median length of stay in the ICU was three days (IQR: 1-5 days), and the median length of hospital stay was six days (IQR: 2-15 days).

**Table 5:** Mortality rate by source of infection in surgical patients with septic shock ICU: intensive care unit

		Survival, n	Death, n	Total, n
Source of infection	Community-onset sepsis	9	40	49
	Hospital-onset sepsis	2	8	10
	ICU-onset sepsis	1	1	2
Total		12	49	61

## Discussion

In this prospective study, 61 surgical patients were identified to have septic shock as per the SEPSIS-III definition. Males were more commonly affected than females, which aligns with studies from Addis Ababa, Ethiopia and Turkey [5,11]. Most of the infections in our study were community-onset sepsis (83.3%), similar to the findings in the Addis Ababa study [5]. For most of the patients in this study, the focus of sepsis was intra-abdominal; however, studies from Germany, Turkey, and Addis Ababa showed the respiratory tract to be the focus of sepsis [5,11-12]. This difference could be explained by the fact that we included only surgical patients and the most common procedure was abdominal surgery. In studies conducted in Sri Lanka and Mexico, the most common site of infection was urine [13,14]. The most common comorbid condition in our study was malignancy followed by hypertension, which contrasts with previous studies [11,13]; most of our patients were from rural areas. HIV was one of the most comorbid conditions in studies done in Africa; the study from Ethiopia reported an HIV comorbidity of 19.3%; [5]. However, in our study, only 13.1% of the patients were screened for HIV due to the unavailability of a testing kit.

Management of septic shock

All patients with septic shock required ICU care due to multiple organ dysfunction (The mean mSOFA score at admission was 10 in our study); 68% of patients with septic shock in our study received mechanical ventilator support compared to the study done in Turkey in which 87.2% of the patients were on mechanical ventilation, due to our limited hospital resources [11]. The SSC one-hour bundle elements include obtaining blood cultures before administering antibiotics, administering broad-spectrum antibiotics, starting appropriate fluid resuscitation, measuring serum lactate, and beginning vasopressors if clinically indicated [1,15]. In this study, the SSC one-hour bundle was as follows - measurement of serum lactate level: 0%; blood cultures obtained before administering antibiotics: 0%; 95.1% of patients received antibiotics within one hour; administration of intravenous fluids; 55.7%; and vasopressor adrenaline was given to 61% of patients within the first six hours. All the patients in our study received antibiotics, and for most patients, the antibiotic choice was empirical and it was given as combination therapy (85.2%).

In the study done in Addis Ababa, 89.5% of patients received antibiotics empirically, while it was combination therapy in 78.8% of patients [5]. Blood cultures should be obtained before the initiation of the antibiotics. In our study, samples were obtained after the initiation of empirical antibiotics. Microbial cultures were obtained from only (18%) of patients, which is lower compared to the study from Addis Ababa, in which microbiological culture samples were obtained from 32.4% of patients [5]; also, a study from Sri Lanka showed that blood cultures taken before antibiotics was 58.1% [13]. This shows that most of the antibiotic therapy in our study was not based on cultures and sensitivity results, and there was no understanding of the need for proper cultures and administration of antibiotics. In this study, adrenaline was the initial vasopressor given. According to the SSC guideline, noradrenaline is the first-line agent to be used for restoring blood pressure and adrenaline can be used if noradrenaline is not available [1].

Measurement of serum lactate level was not done in all the patients due to its unavailability in the facility; lactate level measurement constitutes one of the recommendations in the SSC guidelines 2021, and it is essential for lactate-guided resuscitation, which significantly reduces the high mortality rate associated with elevated lactate levels (>4 mmol/L) [1,3]. Many studies have shown that adherence to the SSC guidelines is effective in managing sepsis and septic shock; however, these studies were mostly conducted in high-income countries [1,16]. A study in Australia showed that SSC bundle compliance was associated with lower mortality and ICU admission rates in patients with sepsis. Still, countries with low resources may experience a different impact [1]. Other studies have shown poor compliance with the SSC one-hour bundle but still with a low mortality rate compared to this study.

Mortality related to septic shock

Patients with septic shock often have a high mortality rate [2,3]. A systematic review and meta-analysis conducted between 2009 and 2019 reported a 30-day septic shock-related mortality of 33.7% (95% CI: 31.5-35.9) in North America, 32.5% (95% CI: 31.7-33.3) in Europe, and 26.4% (95% CI: 18.1-34.6) in Australia [12]. Also, a study from Germany found that the mortality rate for septic shock according to the SEPSIS-III definition was 50.9% [12,17]. While these studies were performed among medical and surgical patients, our study included only adult surgical patients. In our study, the 30-day mortality rate was as high as 80.1%, and the ICU mortality rate was 78 (94%). Similarly, a study in Turkey also showed a high mortality rate: 75% according to the SEPSIS-III definition and 70.4% according to SEPSIS I [11].

The study from Addis Ababa revealed that patients with septic shock had more than twice the mortality rate (75.5%) compared to those without shock (36.4%). The median hospital stay was six days (IQR: 2-15 days), and the ICU stay was three days (IQR: 1-5 days) in our study, due to high early mortality. These findings correlate with the study conducted in Addis Ababa, which reported a median ICU stay of five days (IQR: 2-8 days) [5]. Other studies from high-income countries such as Germany, the United States, and China have shown higher lengths of hospital and intensive care stay, largely due to advanced ICU care and better medical outcomes related to mortality rates in these countries [12,17,18].

Limitations of the study

This study has a few limitations. This study was institutional-based with a small sample size, and the study duration was only six months, a short duration compared to other studies. Moreover, the resources for vital investigations like serum lactate levels or arterial blood gases were not available in the hospital.

## Conclusions

The mortality rate in this study was much higher than in similar studies conducted in high-income and low-income countries. The study revealed poor compliance and adherence to SSC guidelines. More studies should be conducted to gain deeper insights into the challenges encountered in implementing the SSC management guidelines in low-income countries. The hospital and the ICU lengths of stay were lower compared to studies from high-income countries due to high early mortality. Having advanced ICU care and healthcare systems can improve the outcomes related to mortality rates in low-income countries such as ours.
